# Topological control of mechanical resilience and tunable thermal transport in 3D boron nitride honeycombs

**DOI:** 10.1039/d6ra04965f

**Published:** 2026-08-11

**Authors:** Minh-Quan Doan, Tuan-Dung Nguyen, Tuan Nguyen Van, Douglas Soares Galvão, Manh-Huong Phan, Le Van Lich

**Affiliations:** a Center for Materials Innovation and Technology, VinUniversity Hanoi Vietnam lich.lv@vinuni.edu.vn; b Department of Physics, Le Quy Don Technical University Hanoi Vietnam; c Department of Applied Physics, State University of Campinas Campinas São Paulo 13083-970 Brazil; d College of Engineering and Computer Science, VinUniversity Hanoi Vietnam

## Abstract

The rational assembly of low-dimensional building blocks into three-dimensional (3D) architectures offers an exceptional framework for tailoring macroscopic thermal transport while maintaining structural integrity. In this work, we employ non-equilibrium molecular dynamics simulations to systematically investigate the thermal conductivity and mechanical response of topologically porous 3D boron nitride honeycomb (3D-BNHC) nanostructures. Our results reveal that 3D-BNHCs exhibit an ultralow in-plane thermal conductivity below 5 W mK^−1^, representing an unprecedented reduction of approximately two orders of magnitude compared to pristine 2D hexagonal boron nitride. Spectral heat current analysis and spatial flux mapping demonstrate that this severe suppression originates from intense phonon scattering at the monatomic junctions, coupled with localized, closed heat flux trajectories that effectively trap thermal carriers. Furthermore, the continuous yet porous network induces a profound spatial thermal anisotropy. Out-of-plane transport remains highly efficient, scaling linearly with structural density, whereas in-plane propagation obeys a distinct, geometric path-dependent power law characteristic of highly porous macroscopic cellular media. Uniaxial deformation reveals that the network preserves robust structural integrity, displaying stretch-dominated load-bearing capacity under tension and a multi-stage, buckling-driven progressive collapse under compression that provides exceptional energy absorption and mechanical damping. Crucially, this structural flexibility enables robust and reversible dynamic tuning of thermal transport, driven by the competing mechanisms of lattice buckling and transport-axis alignment. Providing a deep microscopic understanding of topology–property relationships, our findings position 3D-BNHCs as premier candidates for extreme-environment thermal insulation, protective mechanical damping, and strain-engineered thermal management systems.

## Abstract 2

3D boron nitride honeycombs exhibit ultralow in-plane thermal conductivity (<5 W mK^−1^) *via* junction phonon scattering. They feature thermal anisotropy, robust energy absorption, and reversible strain-tunable transport.

## Graphical abstract

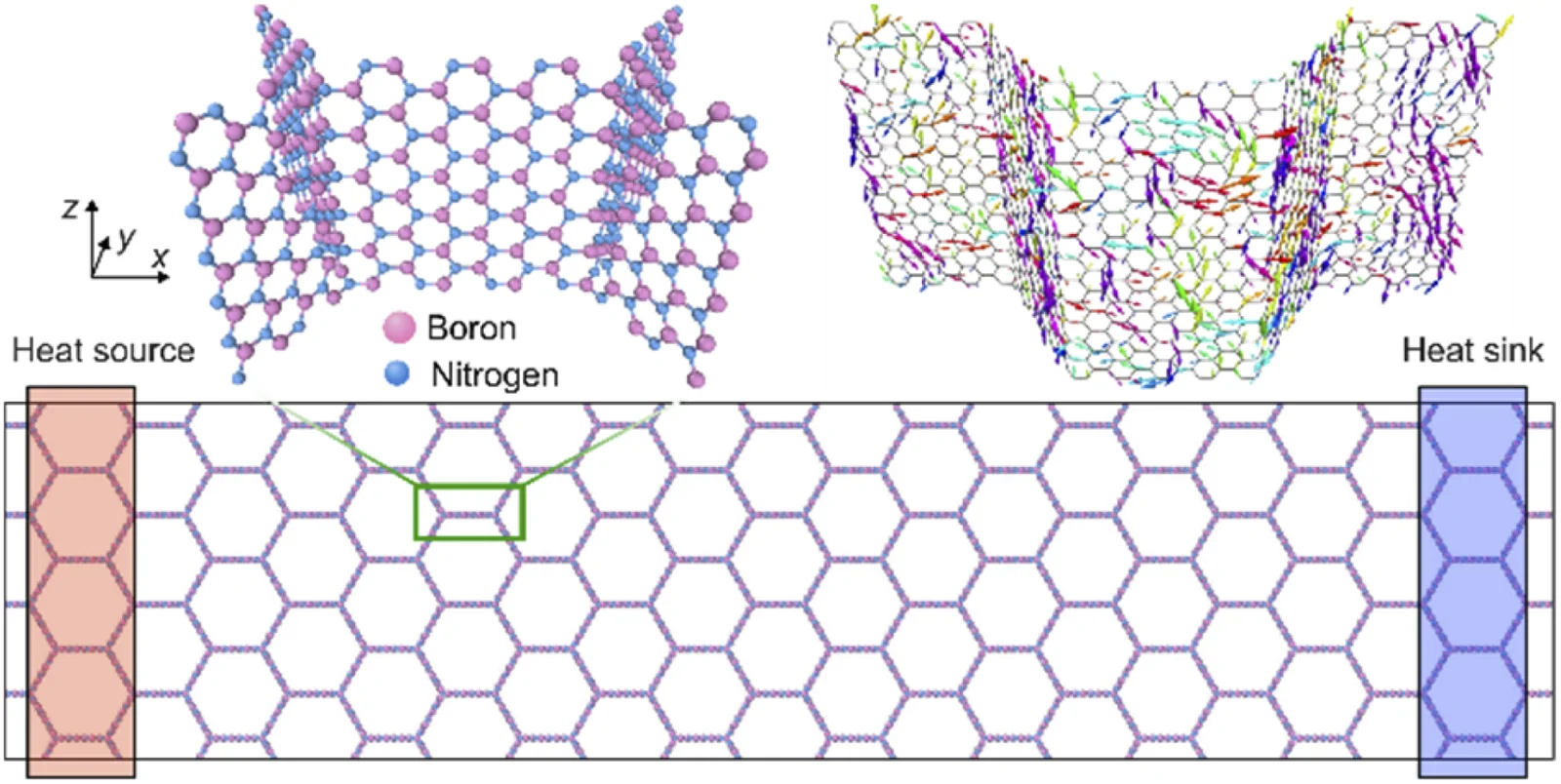

## 1 Introduction

Hexagonal boron nitride (hBN) has emerged as a fundamental two-dimensional (2D) building block due to its remarkable mechanical strength, wide electronic band gap, and high chemical stability.^1–3^ Similar to graphene, hBN possesses an identical honeycomb lattice structure but features alternating boron and nitrogen atoms governed by partially ionic sp^2^ covalent bonds.^3,4^ This unique electronic topology endows hBN with a broad band gap,^4,5^ making it an exceptional electrical insulator while retaining superb structural integrity.^6^ However, while intrinsic hBN exhibits exceptional in-plane thermal conductivity (∼300–400 W mK^−1^ experimentally and up to 750 W mK^−1^ theoretically),^7,8^ its macroscopic utility is often hindered by pronounced lattice anisotropy and the degradation of thermal transport during device integration.^7^ The weak van der Waals interactions between adjacent stacked layers result in an out-of-plane thermal conductivity that is significantly lower than its in-plane counterpart.^9^ This severe directional disparity, combined with high contact resistance at boundaries, poses significant challenges when integrating hBN into modern electronic components, aerospace applications, and composite matrices. These limitations have induced a shift toward structural engineering, at which the research axis transitions from the single sheet to the collective behavior of complex interconnected networks.

The assembly of 2D precursors into three-dimensional (3D) architectures offers a transformative pathway to bridge nanoscale properties with macroscopic functionality.^10–12^ By designing 3D interconnected frameworks, such as aerogels or porous foams, it is possible to leverage the oxidation resistance of hBN while inducing novel characteristics such as ultralow density and high porosity. Unlike carbon-based architectures which face oxidative degradation above 773 K,^13^ hBN structures maintain structural integrity, providing a unique solution for extreme-environment insulation and fire-resistant applications.^14^ In these systems, the transition from a continuous 2D lattice to a porous 3D network fundamentally reconfigures energy transport.^14^ By tailoring the geometric alignment, node geometry, and ligament thickness, researchers can drive these networks into ultralow thermal conductivity regimes that are heavily decoupled from the bulk properties of the parent material. To bridge the gap between nanoscale single-sheet properties and macroscopic functionality, recent experimental efforts have extensively explored boron nitride (BN)-based composite structures and porous architectures for high-performance heat conduction and thermal interface materials. For instance, functionalized hBN combined with liquid metal in flexible polymer matrices creates continuous dual-network pathways, achieving superior thermal conductivity and low interfacial thermal resistance.^15^ Additionally, vertically aligned anisotropic BN nanosheet aerogels prepared *via* directional freezing techniques demonstrate the feasibility of tailoring structural orientation to modulate localized heat conduction.^16^ Other experimental strategies, including surface modification of BN interfaces and advanced microchannel thermal management designs, further highlight the versatility of BN in thermal transport applications.^17^ These practical advances underscore the importance of underlying topological controls and continuous structural networks in optimizing thermal performance across multi-scale BN architectures.

Among these architectures, 3D honeycomb topologies are of particular interest due to their mathematically optimal material distribution and high mechanical energy absorption efficiency.^18^ Inspired by the successful experiment of stable 3D graphene honeycombs *via* chemical vapor deposition and template-directed assembly,^19^ as well as computational evaluations of thermal transport in 3D graphene frameworks,^20^ analogous frameworks based entirely on hBN have been theoretically validated and explored.^21–23^ These architectures are composed of interlinked hBN nanoribbons joined seamlessly at periodic, monatomic triple lines, preserving the robust covalent sp^2^-hybridized bonding environment of their 2D precursors within a high-porosity framework.^21,22^ This keeps the local atomic structure virtually free of dangling bonds, minimizing intrinsic defect states that typically compromise chemical stability. Nevertheless, while the mechanical deformation modes, electronic band-gap modulations, and structural stability of these networks under ambient conditions are increasingly well-characterized,^21,22^ a comprehensive description of steady-state thermal transport in 3D-BNHCs remains elusive. Most existing studies focus primarily on the electronic structure or static mechanical properties, leaving a vital knowledge gap regarding how heat propagates through these complex, interconnected topologies. The intricate interplay between structural density, spatial lattice anisotropy, and mechanical strain is expected to fundamentally redistribute the phonon transport pathways, yielding the ultralow thermal conductivity characteristic of highly porous media. Specifically, how the monatomic junction lines alter the phase space of acoustic phonons and whether the macroscale scaling laws of classical foam mechanics hold true at the nanoscale remain key unresolved questions.

While previous theoretical studies have validated the structural stability and electronic band structures of 3D-BNHC architectures, a comprehensive description of steady-state thermal transport, spatial anisotropy, and dynamic strain tuning remains elusive. In this study, non-equilibrium molecular dynamics (NEMD) simulations are employed to systematically characterize thermal transport and mechanical response in 3D-BNHC nanostructures. Our results reveal that these architectures exhibit ultralow in-plane thermal conductivities, highlighting an unprecedented suppression of heat transport driven by intense phonon scattering at the triple-junction lines and localized, closed heat flux trajectories. Furthermore, density-dependent scaling laws are established along the principal crystallographic axes, uncovering a profound spatial thermal anisotropy where out-of-plane transport scales linearly with density while in-plane propagation obeys a distinct, path-dependent power law. Finally, the influence of uniaxial mechanical strain is clarified, revealing a highly responsive framework for the dynamic, reversible tuning of thermal transport *via* lattice buckling and transport-axis alignment. These findings provide a deep microscopic understanding of topology–property relationships, positioning 3D-BNHCs as exceptional candidates for next-generation thermal management systems.

## 2 Methods

**Fig. 1:**
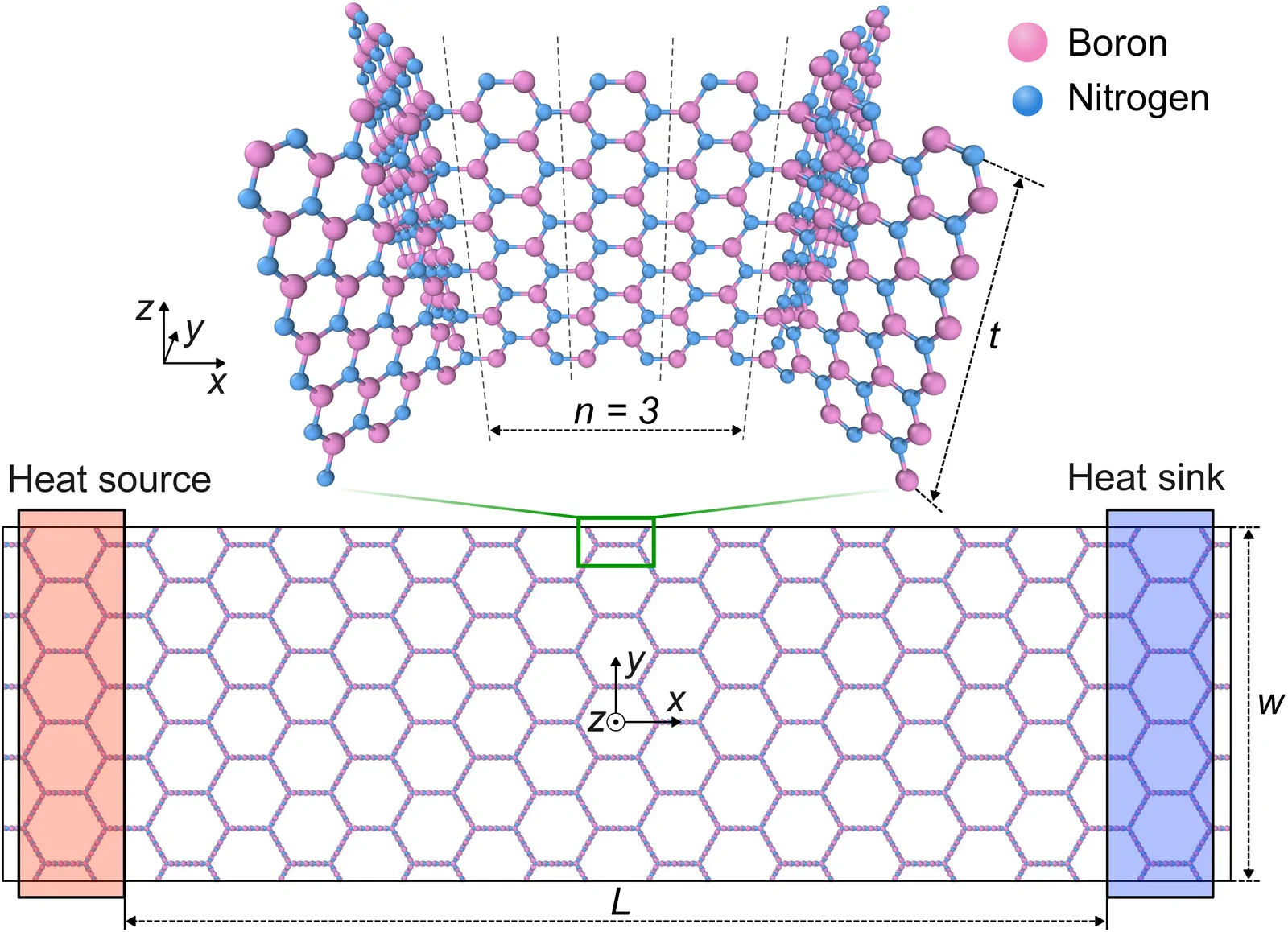
Atomic configuration of the 3D boron nitride honeycomb (3D-BNHC). The top panel shows the perspective view of a unit cell with characteristic width *n* = 3, composed of boron (pink) and nitrogen (blue) atoms. The bottom panel illustrates the non-equilibrium molecular dynamics (NEMD) simulation setup, featuring a sample of length *L* and width *w* connected to a heat source (red) and heat sink (blue) to establish a steady-state temperature gradient.

3D-BNHC models are constructed by seamlessly interconnecting zigzag BN nanoribbons into a honeycomb topology (Fig. 1). The geometry consists of three nanoribbons converging at monatomic junction lines, composed entirely of B or N atoms, and periodically distributed along the *z*-axis.^21–23^ Crucially, all atoms maintain sp^2^ hybridization, preserving the strict alternating covalent BN bonding environment,^24^ which leaves the local atomic structure virtually free of dangling bonds and inherently minimizes pristine defect states. The architecture is defined by six walls of the hBN nanoribbon, with a characteristic width *n* that corresponds to the number of hBN unit cells between adjacent junctions (Fig. 1). By varying *n* from *n* = 1 to *n* = 5, structural models are generated with tunable geometric profiles that systematically expand the internal core pore volume. Furthermore, the volumetric mass density (*ρ*) is inversely proportional to *n*, allowing precise density modulation through adjustment of the characteristic width. To establish a predictive transport framework, these structural changes can be represented *via* the relative density, defined as *ρ*_r_ = *ρ*/*ρ*_0_, where *ρ*_0_ = 2.1 g cm^−3^ is the theoretical density of bulk pristine hBN.^25^ A total of 113 BNHC models were generated, spanning lengths (*L*) of 7.0–82.8 nm, widths (*W*) of 6.82–20.25 nm, and thicknesses (*t*) of 6.89–19.68 nm.

Molecular dynamics (MD) simulations were performed using the high-performance graphics processing units molecular dynamics (GPUMD) package.^26^ This software platform was selected to exploit its highly efficient GPU-accelerated domain decomposition algorithms, which enable the prolonged timescales necessary to reach stable non-equilibrium thermal steady states in complex porous networks. The interatomic interactions governing the covalently bonded B and N atoms were modeled *via* the optimized Tersoff potential parameterized by Lindsay and Broido,^27^ which accurately captures the anharmonic phonon–phonon scattering channels and acoustic branch dispersions in sp^2^-hybridized boron nitride lattices. Note that while the Lindsay–Broido optimized Tersoff potential is highly robust for capturing the harmonic and anharmonic phonon dynamics of sp^2^ B–N networks, we acknowledge that empirical potentials possess inherent limitations in highly non-equilibrium or severely distorted structural states. Recently developed machine learning potentials (MLPs) offer a highly promising alternative by bridging DFT-level accuracy with classical molecular dynamics scales.^28^ While the present potential is quantitatively benchmarked against experimental hBN thermal properties to ensure reliability, incorporating MLPs represents an important avenue for future investigations to more precisely explore extreme multi-axial fracturing and structural phase reconstructions in BNHCs. Periodic boundary conditions were strictly applied in all three principal spatial directions to eliminate non-physical edge relaxation effects. To establish a physically realistic starting configuration at ambient conditions, the systems were first subjected to an exhaustive equilibration protocol at 300 K. This involved sequential relaxation under the isothermal-isobaric (NPT) ensemble^29^ for 200 ps to optimize cell dimensions and eliminate residual axial stresses, followed by a canonical (NVT) ensemble^30^ run for 200 ps, and a final microcanonical (NVE) ensemble phase to guarantee total energy conservation. The classical equations of motion were numerically integrated using the velocity Verlet algorithm with a well-tested timestep of 0.5 fs. The results of the convergence tests are presented in Fig. S2 of the SI.

Thermal conductivity (*κ*_*ij*_) was systematically evaluated using the non-equilibrium molecular dynamics (NEMD) method by imposing a steady-state heat flux along the specific transport direction under investigation (*x*, *y*, or *z*), as schematically illustrated in Fig. 1. To establish a well-defined temperature gradient across the transport axis, fixed boundary conditions were enforced by freezing monatomic atomic layers at both extreme ends of the simulation domain, thereby preventing translational slipping and suppressing unphysical boundary reconstruction. Immediately adjacent to these rigid boundaries, local heat source and sink regions of equal width were defined. A constant, stable macroscopic temperature gradient of 0.15 K Å^−1^ was established and dynamically regulated by applying local Langevin thermostats^31,32^ to these boundary zones, which continuously modulated the kinetic energy to maintain the source and sink at *T* + Δ*T*/2 and *T* − Δ*T*/2, respectively.

The extensive intermediate region containing the active 3D-BNHC network between the source and sink was allowed to freely evolve under the microcanonical (NVE) ensemble, ensuring that energy transport was governed entirely by intrinsic lattice dynamics without deterministic thermostat artifacts. Following an initial 500 ps non-equilibrium equilibration period to allow the thermal wavefront to cross the domain and stabilize the spatial temperature profile, a subsequent 500 ps production run was conducted. This duration ensured rigorous steady-state heat transfer, verified by tracking the strict linearity of the spatial temperature gradient (Δ*T*/*L*) across the active region and checking the energy conservation balance between the hot and cold reservoirs. The directional thermal conductivity was then calculated *via* the direct application of Fourier's law:^33^1
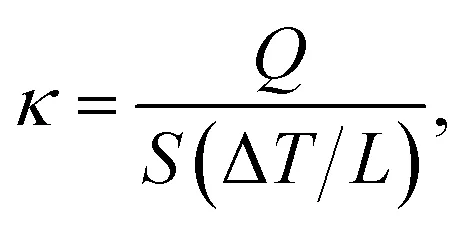
where *Q* = Δ*E*/Δ*t* is the time-averaged heat flow rate (with Δ*E* representing the cumulative energy added to the source and extracted from the sink over the steady-state time interval Δ*t*), *S* is the effective cross-sectional area perpendicular to the heat flux, and *L* denotes the precise distance separating the centroids of the heat source and sink regions. Finally, to uncover the underlying microscopic mechanisms governing the thermal transport reduction in these porous topologies, spectral heat current (SHC) analysis was executed using the virially derived heat flux operators implemented within the GPUMD framework. This allowed the decomposition of thermal transport into frequency-resolved components, successfully isolating the role of acoustic phonon modes and intensive junction-induced phase-boundary scattering.

## 3 Results and discussion

### 3.1 Thermal conductivity of 3D boron nitride honeycombs

To rigorously validate the computational framework, accuracy of the chosen interatomic potentials, and reliability of the simulation parameters, the thermal conductivity of monolayer 2D hBN was systematically evaluated as a benchmarking reference prior to exploring the 3D honeycomb architectures. Given that NEMD simulations are inherently subject to pronounced finite-size effects arising from truncation of long-wavelength phonon modes, we executed simulations across a wide array of system lengths. Utilizing a standard linear extrapolation scheme based on the inverse relationship between the calculated thermal conductivity (1/*κ*) and the simulation domain length (1/*L*), the thermodynamic limit for an infinite sample length was successfully isolated. Our extrapolated bulk thermal conductivity value for the pristine 2D hBN sheet is determined to be 737 W mK^−1^ (Fig. S1 in the SI). This calculated value exhibits exceptional agreement with the high-temperature experimental benchmark of 751 W mK^−1^ reported in the previous study.^8^ This high degree of quantitative consistency robustly substantiates the accuracy of our NEMD protocol, confirming its competency to capture the underlying phonon transport mechanics and accurately predict the thermal properties of highly complex, topologically modified BNHC nanostructures.

**Fig. 2:**
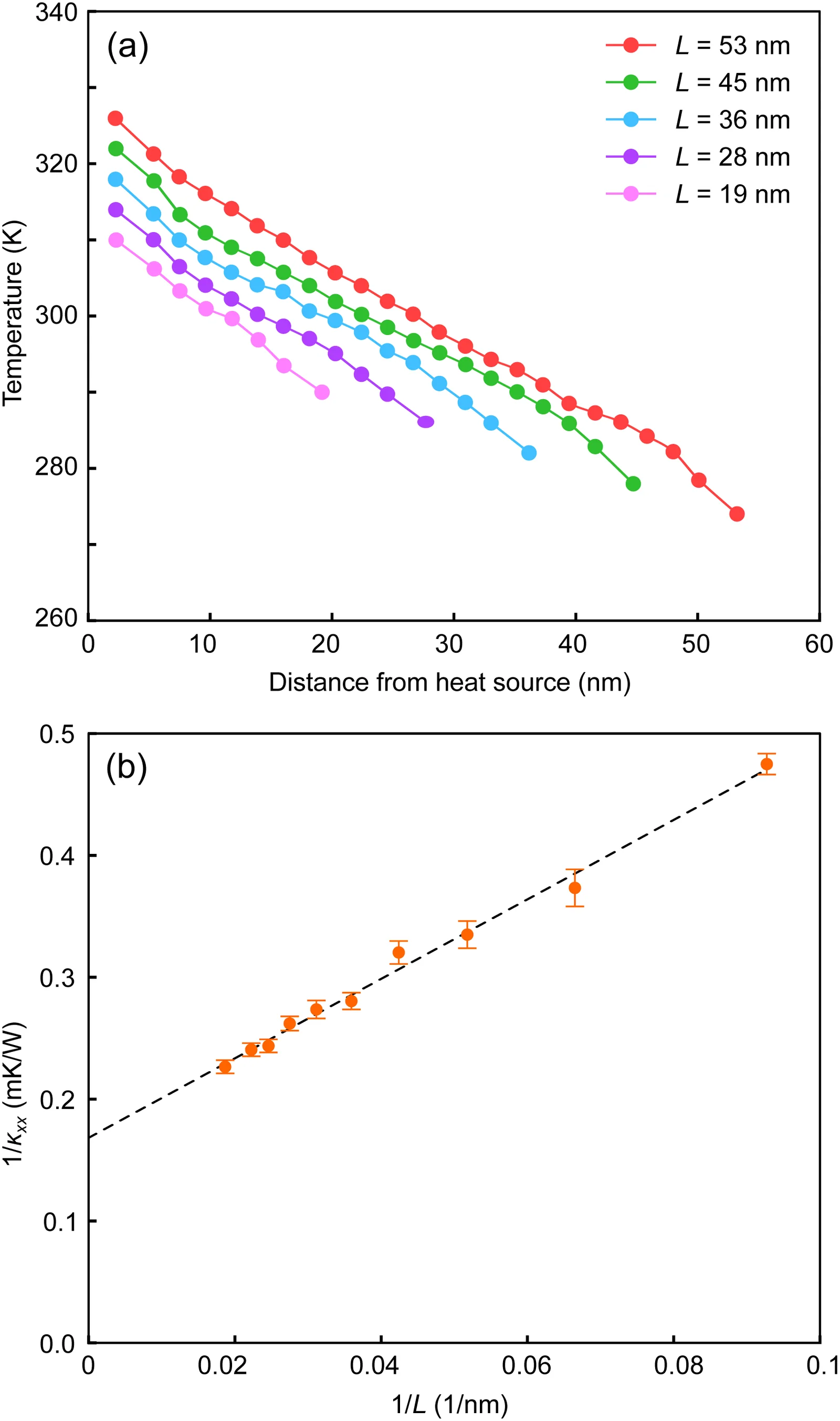
(a) Steady-state temperature distribution along the transport direction for BNHCs with lengths (*L*) ranging from 10 nm to 42 nm, showing linear gradients. (b) Extrapolation of bulk thermal conductivity using the linear relationship between inverse thermal conductivity (1/*κ*_*xx*_) and inverse system length (1/*L*).

Thermal transport in the 3D-BNHCs was first systematically investigated by applying a steady-state heat flux along the *x*-direction. Representative temperature profiles for BNHCs with a characteristic width of *n* = 3 across varying sample lengths (*L*) are shown in Fig. 2(a). All calculated temperature profiles exhibit remarkably linear gradients between the heat source and sink, which confirms that a stable, non-equilibrium steady-state heat transport regime was fully established during the simulations. As expected, longer samples demonstrate progressively reduced temperature gradients. This behavior reflects a corresponding change in the effective thermal resistance of the nanostructures, dictated by the increased distance over which phonon modes undergo scattering and energy dissipation. The NEMD-derived thermal conductivity (*κ*_*xx*_) is typically subjected to finite-size effects when the simulation domain length (*L*) is comparable to or smaller than the intrinsic mean free path (MFP) of the dominant phonons.^34^ Under these conditions, phonons with long mean free paths propagate ballistically across the active region without undergoing full intrinsic phonon–phonon scattering. Instead, their transport is artificially truncated by non-physical scattering at the thermostat boundaries, which imposes a size-dependent boundary thermal resistance that artificially lowers the calculated apparent thermal conductivity *κ*(*L*). To eliminate these finite-size boundary artifacts and extrapolate the true, size-independent bulk thermal conductivity (*κ*_∞_), we simulated ten different lengths for each characteristic width *n* (as compiled in Table S1 in the SI). We then exploited the established inverse linear scaling relationship between the inverse thermal conductivity (1/*κ*_*xx*_) and the inverse system length (1/*L*). As illustrated in Fig. 2(b), the collected data points exhibit a robust linearity across all modeled lengths. This exceptional linear fit justifies our computational domain sizing and enables a highly precise, thermodynamic-limit extrapolation toward an infinite system length (1/*L* → 0). By capturing the intercept of these linear regressions, the bulk intrinsic thermal transport properties of the 3D porous networks were successfully decoupled from arbitrary simulation size artifacts, establishing a rigorous foundation for evaluating structural density and anisotropy effects.

**Fig. 3:**
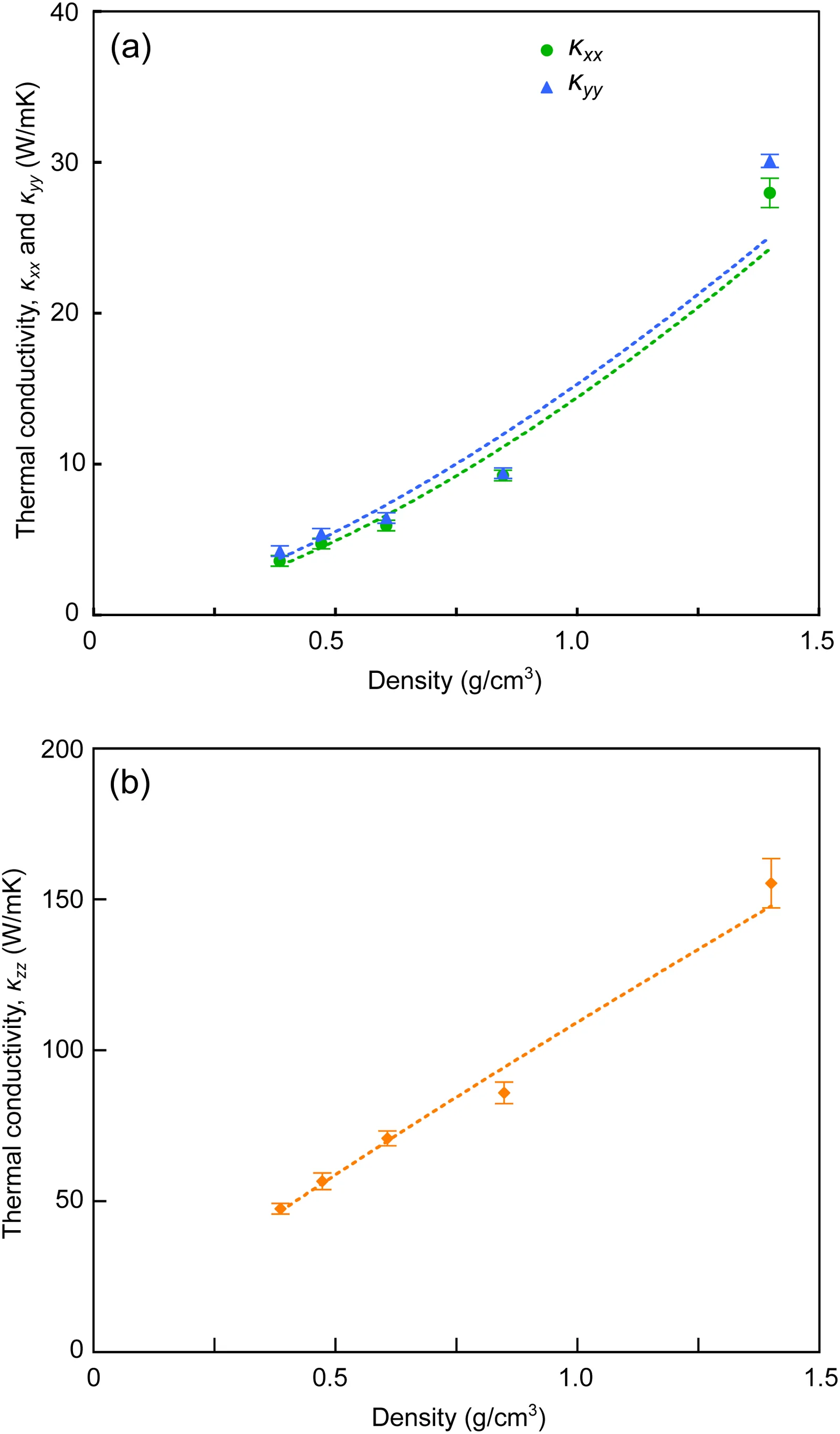
(a) Bulk thermal conductivity along the *x* (*κ*_*xx*_) and *y* (*κ*_*yy*_) directions as a function of material density. (b) Thermal conductivity along the *z* direction (*κ*_*zz*_) *versus* density.

The explicit dependence of the extrapolated bulk thermal conductivity on the structural mass density along the three principal axes (*x*, *y*, and *z*) provides crucial insight into the lattice configurations of the 3D-BNHC networks. As illustrated in Fig. 3(a), the in-plane thermal conductivities (*κ*_*xx*_ and *κ*_*yy*_) are highly sensitive to variations in structural density. By systematically tuning the characteristic wall width (*n*) from *n* = 1 to *n* = 5, the pore volume expands, enabling direct modulation of the volumetric density. This architectural reconfiguration induces a dramatic, monotonic decline in both *κ*_*xx*_ and *κ*_*yy*_, which converge to an ultralow regime of approximately 5 W mK^−1^ at the lowest density limits. To put this into perspective, this value is roughly two orders of magnitude lower than that of pristine single-layer 2D hBN. This extreme suppression highlights the capability of the 3D porous honeycomb topology to sever long-range phononic paths.

In sharp contrast, thermal transport along the out-of-plane *z*-direction proves to be vastly more efficient, as plotted in Fig. 3(b). The *κ*_*zz*_ values remain significantly higher across all evaluated structural configurations, revealing a pronounced spatial thermal anisotropy within the 3D-BNHC structures. Because the continuous zigzag nanoribbons remain unbroken along the *z*-axis, phonons propagate without encountering structural disruptions. Conversely, in-plane transport along the *x*- and *y*-axes requires heat carriers to navigate across the interconnected junction zones, inducing intensive phase-boundary scattering that severely degrade global propagation.

To establish a predictive framework, these directional thermal behaviors were modeled as a function of the relative density, defined as *ρ*_r_ = *ρ*/*ρ*_0_, where *ρ*_0_ = 2.1 g cm^−3^ is the theoretical density of bulk pristine hBN.^25^ Across all three dimensions, the thermal conductivity tightly follows a macroscopic power-law scaling relation:^35^

2 *κ* = *Cρ*_r_^*α*^,

where *C* is a structural coefficient and *α* is the scaling exponent. This power-law behavior closely mirrors the transport physics observed in highly porous macroscopic cellular materials, open-cell foams, and low-density aerogels.

Our numerical regressions reveal that the in-plane scaling obeys the profiles *κ*_*xx*_ = 45.56*ρ*_r_^1.55^ and *κ*_*yy*_ = 45.57*ρ*_r_^1.47^. The non-linear exponents (*α* ≈ 1.5) mathematically capture how the geometric complexity of the heat flow path and junction scattering scale as the honeycomb cells expand. Conversely, the out-of-plane scaling is nearly linear, fitting the expression *κ*_*zz*_ = 212.31*ρ*_r_^0.90^. The near-unity scaling exponent (*α* = 0.90) confirms that out-of-plane heat conduction depends linearly on the structural mass density, where the continuous cell walls behave as isolated parallel thermal pathways. Ultimately, these distinct scaling exponents and the order-of-magnitude variations in *C* quantify the pronounced, topology-induced thermal anisotropy inherent to the 3D-BNHC network.

To provide a deeper theoretical foundation for the observed power-law behavior, the distinct directional scaling exponents can be rationalized through structural transport pathways and geometric conduction models. The near-unity out-of-plane exponent (*α* = 0.90) closely conforms to a classical parallel-conduction framework, dictated by the unbroken, continuous zigzag BN nanoribbons running parallel to the *z*-axis. In this orientation, phonons propagate through continuous solid channels free from structural nodes, meaning the effective out-of-plane thermal conductivity (*κ*_*zz*_) scales linearly with the cross-sectional area fraction of the solid skeleton (*κ*_*zz*_ ∝ *ρ*_r_1.0). The minor sub-unity deviation (0.90) reflects weak, localized scattering fields induced by sub-nanometer lattice distortions at the periodic triple lines. Conversely, the in-plane exponents (*α* ≈ 1.5) govern a highly complex, path-dependent transport network. As the characteristic cell width expands and structural density decreases, the geometric complexity and effective path length for in-plane thermal carriers increase substantially. This geometric constraint is severely compounded by the monatomic triple-line junctions. As demonstrated by the spectral heat current analysis and the localized, closed trajectories in the heat flux vector maps, these junctions act as intense phase-boundary bottlenecks that effectively trap and scatter thermal carriers. While classical macroscopic open-cell foams^36^ experiencing purely geometric path reduction typically yield thermal exponents between 1.5 and 2.0, our nanoscale exponent of *α* ≈ 1.5 mathematically quantifies the combined impact of structural tortuosity and frequency-dependent boundary scattering at the periodic junctions as the porous network scales.

**Fig. 4:**
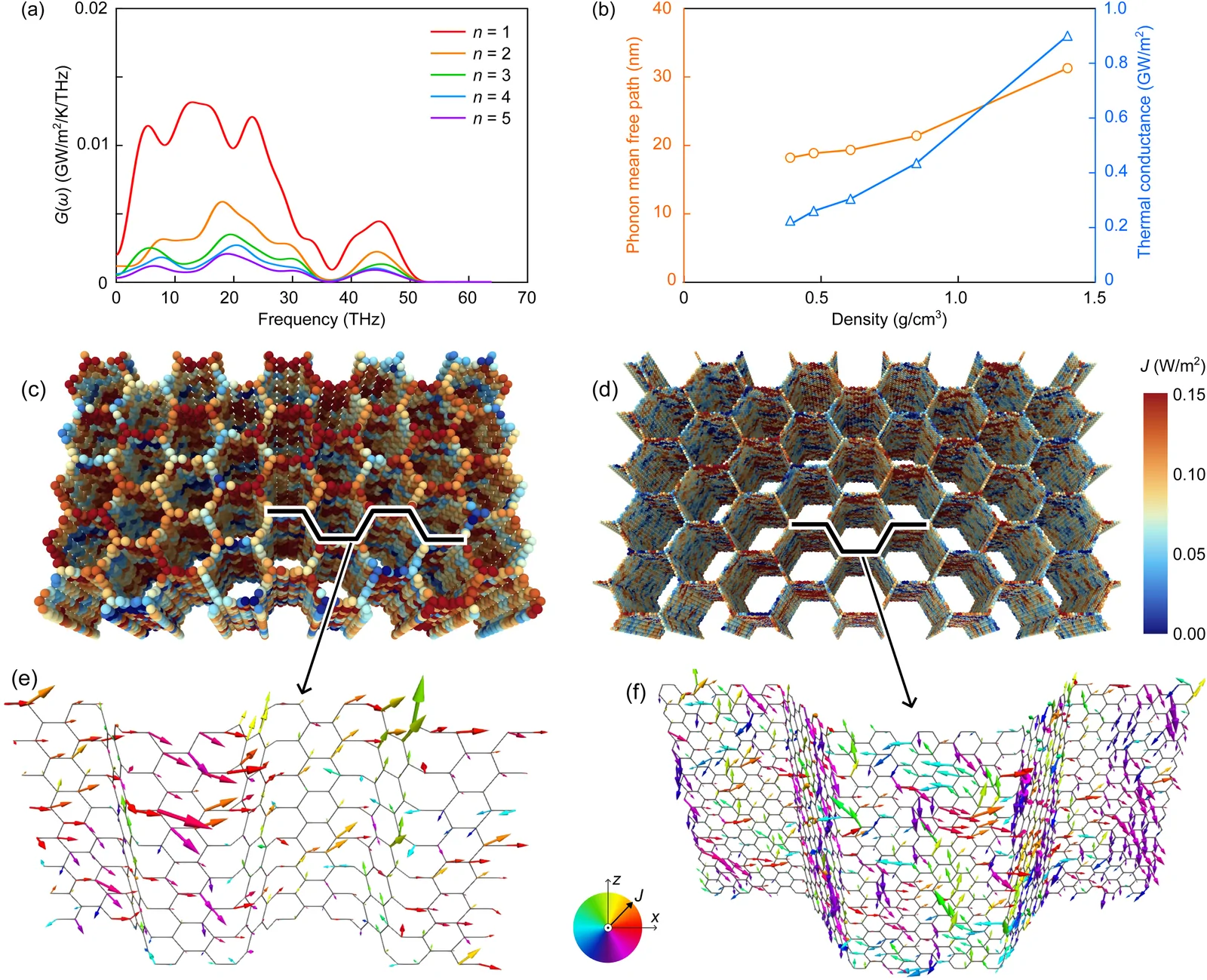
(a) Spectral ballistic thermal conductance (*G*(*ω*)) as a function of phonon frequency for different characteristic widths (*n* = 1 to 4). (b) Variation of the phonon mean free path and total thermal conductance with relative material density. (c and d) Spatial distribution of atomic heat flux magnitude for models with *n* = 1 and *n* = 4, respectively. (e and f) Vector maps of heat flux direction.

Spectral ballistic thermal conductance (*G*(*ω*))^37^ reveals prominent size effects and structural dependencies in 3D-BNHCs (Fig. 4(a)). The spectral analysis indicates that low-frequency phonons spanning from 0 to 30 THz dominate the *G*(*ω*) across all configurations, highlighting the crucial role played by acoustic modes in transporting heat through these networks. The peak of the spectral conductance increases markedly as the characteristic nanoribbon wall width *n* decreases. This enhancement occurs because a smaller *n* corresponds to a higher relative structural density, providing more solid transport channels per unit volume. Additionally, slight frequency shifts in the conductance peaks are observed at larger *n*, indicating that expanding the honeycomb cell size subtly modulates the underlying phonon group velocities and acoustic branch characteristics. When integrated over the entire frequency spectrum, the macroscopic consequences of these nanoscale variations become evident. Both the total thermal conductance and the intrinsic phonon mean free path (MFP) exhibit a concurrent decline as the structural density of the BNHCs decreases (Fig. 4(b)). This trend indicates that the expanding empty core volume in lower-density structures imposes strict geometric boundaries that reduce phonon propagation. The contraction of the MFP reflects an increasingly localized phonon domain where heat carriers are heavily scattered, ultimately suppressing the overall in-plane thermal conductivity.

To further elucidate the microscopic mechanisms underpinning this severe suppression of heat transport, the spatial distribution of the local heat flux vector field is evaluated. The local heat flux (*J*_i_ = −*W*_i_ × *v*_i_)^37^ is expressed as a tensor product of the virial tensor (*W*_i_) and the atomic velocity vector (*v*_i_). The spatial distributions of the atomic heat flux magnitude for BNHCs with *n* = 1 and *n* = 4 are illustrated in Fig. 4(c and d), respectively. Comparing the two structures reveals that the heat flux magnitude is significantly higher and more uniform throughout the framework for *n* = 1, consistent with its higher thermal conductivity. While the local fluxes align structurally with the covalent BN lattice directions in both scenarios, their global alignment and spatial coherence differ fundamentally. This distinction is clearly captured by the detailed vector maps shown in Fig. 4(e and f). For the narrow-walled network (*n* = 1), the flux vectors remain well-ordered and closely follow the primary macroscopic transport axis. Conversely, the expanded network (*n* = 4) exhibits highly disturbed, localized, closed heat flux patterns around the interconnected junction zones. This behavior indicates that phonons traversing the larger honeycomb structures become trapped or diverted into local circular flows around the cells. This highly disturbed flux alignment, combined with intensive scattering at the triple-line junctions, creates a severe bottleneck for global energy propagation. These combined microscopic phenomena explain the drastic reduction in thermal conductivity, positioning the topologically porous 3D-BNHC network as a far more effective thermal insulator than conventional planar 2D systems containing standard point defects.

### 3.2 Mechanical properties

**Fig. 5:**
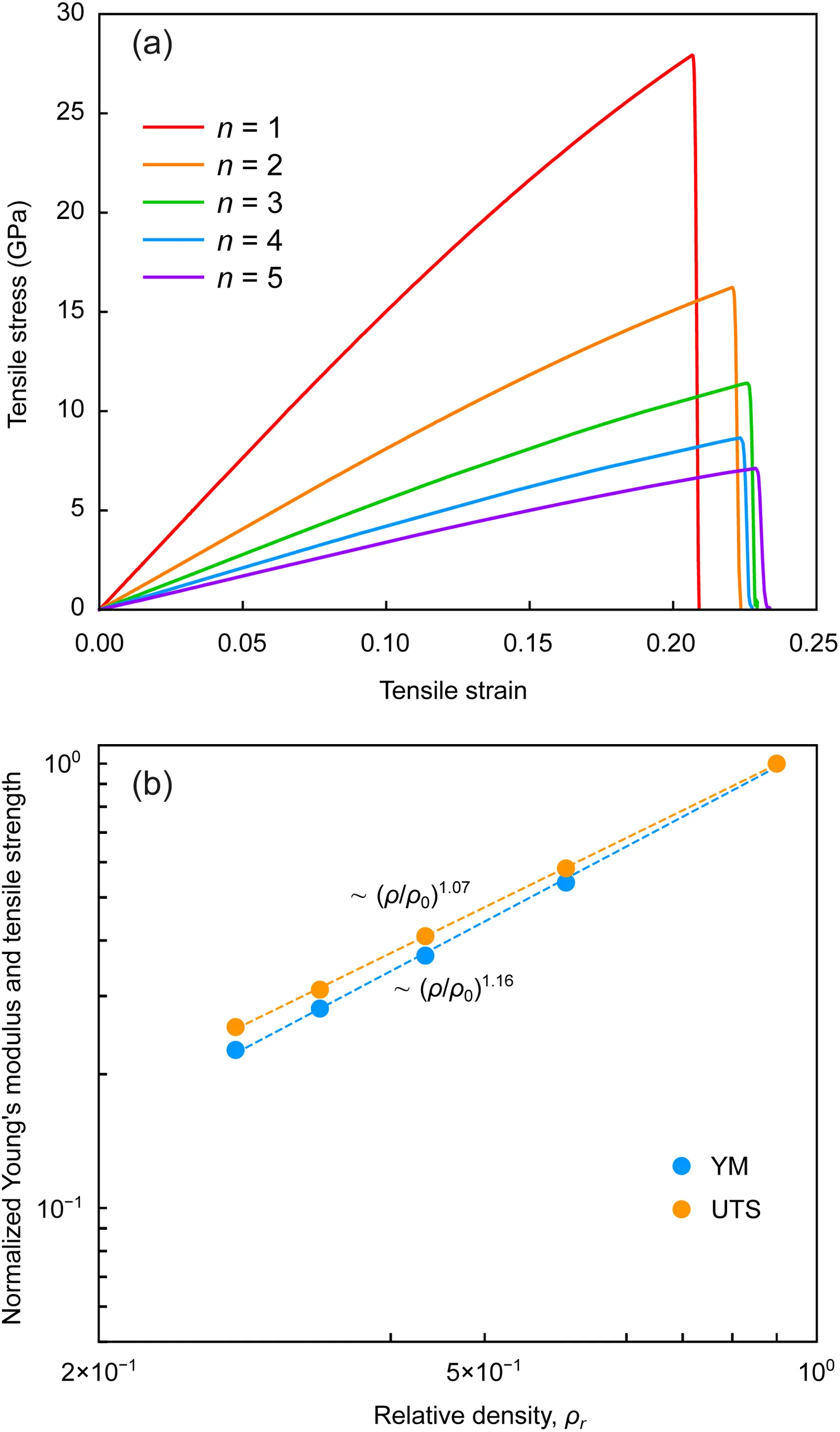
Tensile properties and scaling laws of 3D-BNHC architectures: (a) uniaxial tensile stress–strain curves as a function of characteristic wall width *n*. (b) Power-law scaling behavior of the normalized Young's modulus (YM) and ultimate tensile strength (UTS) relative to the normalized mass density.

Fig. 5 presents the mechanical response of 3D-BNHCs under uniaxial tensile loading along the *x*-direction. Fig. 5(a) illustrates the tensile stress–strain curves for different characteristic widths (*n* = 1 to 5), corresponding to varied relative mass densities. Across all structural configurations, the material exhibits a nearly linear elastic behavior up to a brittle catastrophic failure point. There is a pronounced dependence on the characteristic width (*n*), where the Young's modulus (YM) and ultimate tensile strength (UTS) decrease dramatically as *n* increases from 1 to 5. Specifically, the densest network (*n* = 1) reaches a peak tensile stress of approximately 28 GPa at a failure strain of ∼0.21. As the cell width expands to *n* = 5, lowering the overall material density, the maximum tensile stress falls below 10 GPa, while the fracture strain remains relatively uniform between 0.21 and 0.23. To evaluate how the mechanical integrity scales with structural configuration, Fig. 5(b) plots the YM and UTS as a function of the relative density (*ρ*/*ρ*_0_). Both mechanical parameters demonstrate robust linear relationships on a logarithmic scale, validating macroscopic power-law scaling behaviors. The scaling exponent for the Young's modulus (YM) is determined to be 1.16 (∼(*ρ*/*ρ*_0_)^1.16^), whereas the scaling exponent for the UTS is 1.07 (∼(*ρ*/*ρ*_0_)^1.07^).

The tensile results reveal that 3D-BNHC architectures preserve the outstanding intrinsic mechanical strength of the underlying 2D hexagonal boron nitride building blocks while introducing lightweight, porous characteristics. The brittle fracture mechanism observed uniformly across all curves signifies that failure is dominated by the simultaneous rupture of B–N bonds once the lattice extension limits are reached, showing no signs of plastic deformation. The drastic reduction in both stiffness and UTS at larger values of *n* is fundamentally connected to the reduction of effective load-bearing cross-sectional areas per unit volume. As *n* increases, the empty core volume expands, reducing the structural mass density. This structural dependency follows a predictable power-law behavior. Remarkably, the scaling exponents obtained for YM (1.15) and UTS (1.07) are notably close to 1.0. According to the classical Gibson–Ashby model for cellular solids, an exponent near 1.0 is indicative of an ideal stretch-dominated honeycomb morphology under axial loading, where structural struts primarily undergo pure tension rather than bending or buckling. This confirms that the periodic triple-line junctions effectively distribute the applied stress throughout the interconnected nanoribbon walls. These lightweight, high-strength characteristics position 3D-BNHCs as exceptional candidates for structural, protective, or thermal insulation applications where keeping weight to a minimum is paramount.

**Fig. 6:**
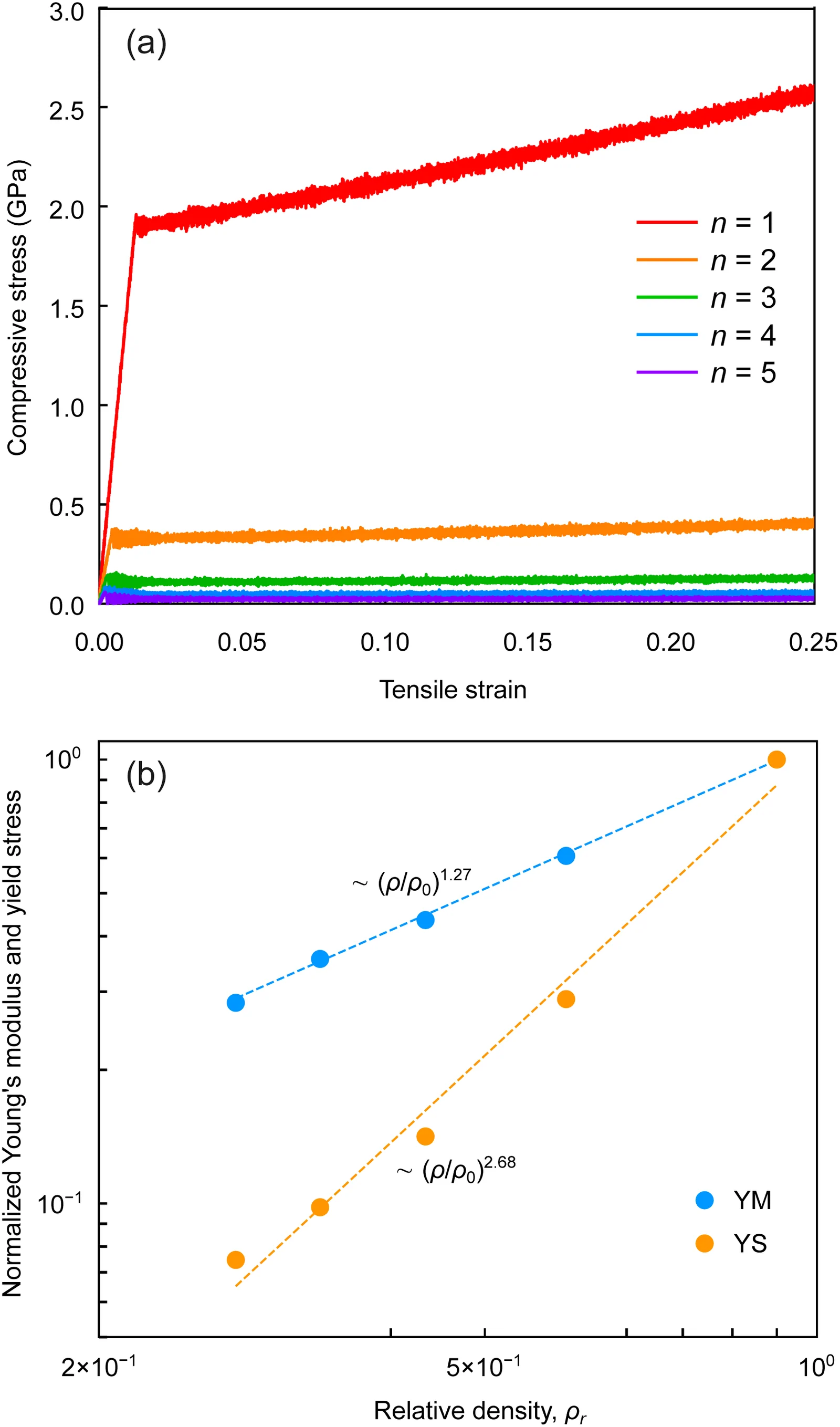
Compressive behavior and scaling laws of 3D-BNHC architectures: (a) uniaxial compressive stress–strain curves as a function of characteristic wall width *n*. (b) Power-law scaling behavior of the normalized compressive Young's modulus (YM) and yield strength (YS) as a function of normalized density.

Fig. 6 presents the mechanical response and corresponding scaling behaviors of the 3D-BNHC architectures under uniaxial compressive loading along the *x*-direction. Fig. 6(a) illustrates the compressive stress–strain curves for the varying characteristic widths (*n* = 1 to 5) up to a strain of 0.25. Unlike the brittle fracture observed under tensile loading, the compressive profiles exhibit a classic multi-stage deformation behavior characteristic of cellular structures. For the densest network (*n* = 1), the stress increases rapidly and linearly within the elastic regime, reaching an initial peak yield stress (YS) of approximately 2.2 GPa at a strain of ∼0.02. Beyond this peak, the curve displays a sudden stress drop followed by pronounced, high-frequency stress oscillations between 1.7 GPa and 2.2 GPa, which gradually transitions into a steady plateau region where stress climbs continuously up to 2.5 GPa at a strain of 0.25. In contrast, the expanded structures (*n* = 2 to 5) demonstrate a massive reduction in compressive strength. For instance, the *n* = 2 structure yields at a much lower stress of ∼0.5 GPa, while the lowest-density networks (*n* = 4 and 5) show nearly flat plateau profiles below 0.2 GPa across the entire strain range. To quantify the structural dependency under compression, Fig. 6(b) plots the logarithm of the YM and YS against the relative density. The data points conform to excellent linear fits, substantiating power-law relationships under compressive conditions. The scaling exponent for the compressive YM is calculated to be 1.27 (∼(*ρ*/*ρ*_0_)^1.27^), whereas the scaling exponent for the compressive YS is determined to be 2.68 (∼(*ρ*/*ρ*_0_)^2.68^).

The distinct features of the compressive stress–strain curves highlight a fundamental transition in the microscopic deformation mechanisms compared to the tensile response. The initial elastic regime represents the uniform elastic bending and compression of the interconnected nanoribbon walls. The subsequent abrupt stress drop and prominent oscillations observed in the higher-density network (*n* = 1) are indicative of localized structural buckling events and successive layer-by-layer collapse of the honeycomb cells. For the lower-density networks (*n* ≥ 2), the structural walls are geometrically more prone to instability, causing them to undergo smooth, continuous cooperative buckling at significantly lower stress thresholds. This explains the absence of severe stress drops and the appearance of long, flat plateau regions, which represent efficient energy absorption *via* progressive cell wall folding without catastrophic material rupture. The extracted scaling exponents provide critical insights into the structural configuration of 3D-BNHCs under compressive stress. The YM exponent of 1.27 lies between the theoretical limits of pure stretch-dominated (1.0) and pure bending-dominated (2.0) cellular structures defined by the classical Gibson–Ashby model. This indicates a mixed mode of deformation where both axial compression and wall bending contribute to the initial structural stiffness. Crucially, the compressive yield strength exponent of 2.08 aligns remarkably well with the ideal Gibson–Ashby scaling law (∼*ρ*_*r*_^2^) expected for conventional cellular solids undergoing plastic collapse or elastic wall buckling. This exponent of ∼2.0 signifies that the ultimate load-bearing capacity under compression is strictly governed by the geometric buckling resistance of the nanoribbon struts rather than the intrinsic material strength of the 2D sheets. This sensitive density-dependent mechanical behavior, combined with the smooth plateau regimes, highlights the capability of 3D-BNHCs to serve as highly tailorable, lightweight energy-absorbing materials and protective acoustic or mechanical dampers.

**Fig. 7:**
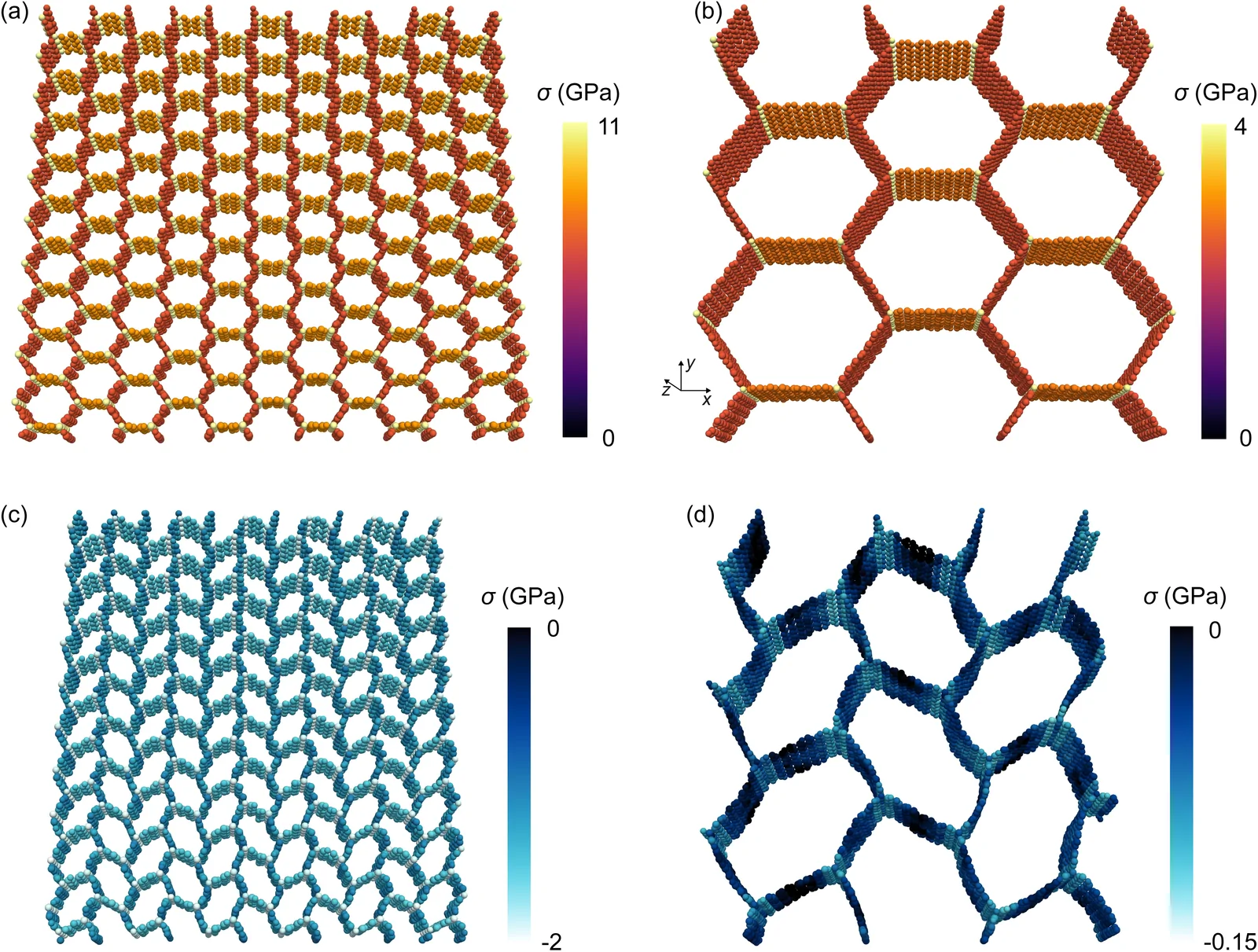
Stress distribution and structural deformation of 3D-BNHC networks with characteristic widths *n* = 1 and *n* = 4 under (a and b) uniaxial tensile strain and (c and d) compressive strain.

Fig. 7 provides a detailed visualization of the atomic-scale stress distribution and progressive structural deformation profiles for the 3D-BNHC networks under varying mechanical loads. The figure compares two distinct architectural configurations: a high-density, narrow-walled network (*n* = 1) and an expanded, lower-density network (*n* = 4). Fig. 7(a and b) illustrate the response of these structures under uniaxial tensile strain applied along the *x*-direction, while Fig. 7(c and d) depict their behavior under uniaxial compressive strain along the same axis. The localized atomic stress values are represented *via* a color-coded gradient, facilitating a qualitative assessment of stress concentration zones, lattice stability, and the specific failure pathways associated with different structural densities.

The structural deformation profiles captured in Fig. 7 visually substantiate the macroscale scaling laws and microscopic mechanical behaviors observed in the quantitative stress–strain analysis. Under uniaxial tensile loading, both the *n* = 1 and *n* = 4 networks display a highly uniform distribution of tensile stresses across the interconnected nanoribbon walls. This uniform pattern confirms a stretch-dominated deformation mechanism, aligning with the classical Gibson–Ashby model for ideal cellular solids where structural struts undergo pure tension. In the dense *n* = 1 network, stress is tightly distributed along the continuous covalent B–N bonds, allowing the structure to maintain a high ultimate tensile strength. In contrast, the expanded *n* = 4 network shows a reduction in load-bearing capacity as stress is concentrated on a scarcer number of solid pathways due to the expanded pore volume. The eventual failure in both cases is a brittle rupture that occurs when the lattice extension limits of the B–N bonds are reached simultaneously.

Under uniaxial compression, the deformation behavior transitions significantly, as the networks shift from stretch-dominated resistance to a multi-stage, buckling-driven progressive collapse. In the high-density *n* = 1 configuration, the walls initially resist the load elastically but eventually undergo sudden, localized structural buckling that results in a layer-by-layer collapse of the honeycomb cells. This process is visualized as abrupt localized distortions and corresponds to the high-frequency stress oscillations observed in the compressive data. Conversely, the lower-density *n* = 4 network features expanded struts that are geometrically more prone to elastic instability. These walls undergo smooth, continuous, and cooperative buckling at lower stress thresholds, enabling the structure to fold progressively into the vacant pore space. This visual mode explains the appearance of long, flat stress plateau profiles, positioning these low-density 3D-BNHC topologies as highly effective candidates for lightweight, rupture-free mechanical energy absorption and impact damping applications. It should be noted that while the network exhibits brittle fracture under uniaxial tension due to simultaneous B–N bond cleavage, its structural resilience and energy dissipation capacity are predominantly realized under compressive loading *via* progressive, non-catastrophic cell-wall folding.

**Fig. 8:**
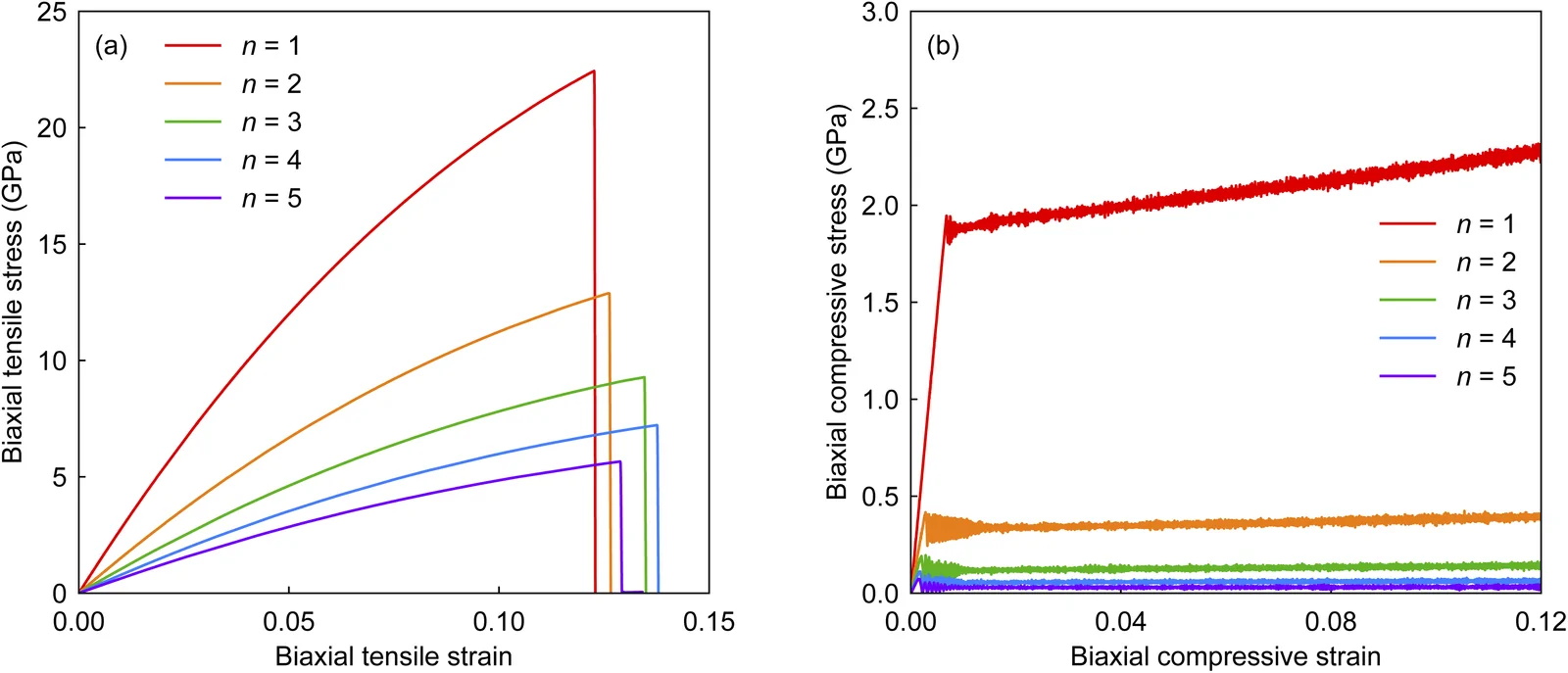
Mechanical response of 3D-BNHC architectures under biaxial loading. (a) Biaxial tensile stress–strain profiles as a function of characteristic wall width (*n*), showing brittle catastrophic fracture under multi-axial extension. (b) Biaxial compressive stress–strain curves, demonstrating a transition to multi-stage cellular collapse characterized by initial yield, stress oscillations, and steady plateau regimes.

Under biaxial loading, the 3D-BNHC architectures maintain deformation mechanics similar to their uniaxial responses while displaying distinct multi-axial stress limits and failure thresholds. Under biaxial tensile strain (Fig. 8(a)), all configurations exhibit linear elastic behavior up to catastrophic brittle fracture, with ultimate tensile strengths decreasing systematically from approximately 23 GPa (*n* = 1) to 6 GPa (*n* = 5) at critical failure strains near 0.13–0.14. Conversely, under biaxial compressive loading (Fig. 8(b)), the frameworks transition to a classic multi-stage cellular collapse. The densest network (*n* = 1) reaches an initial peak yield stress of ∼2.3 GPa prior to characteristic stress oscillations and a rising stress plateau, whereas lower-density configurations (*n* ≥ 2) yield at substantially reduced stresses (≤0.7 GPa) and maintain long, stable stress plateaus across extended strains. This structural response highlights the capacity of 3D-BNHC topologies to accommodate progressive, non-catastrophic cell-wall buckling and energy dissipation under multi-axial compressive conditions.

### 3.3 Strain effect on thermal conductivity

**Fig. 9:**
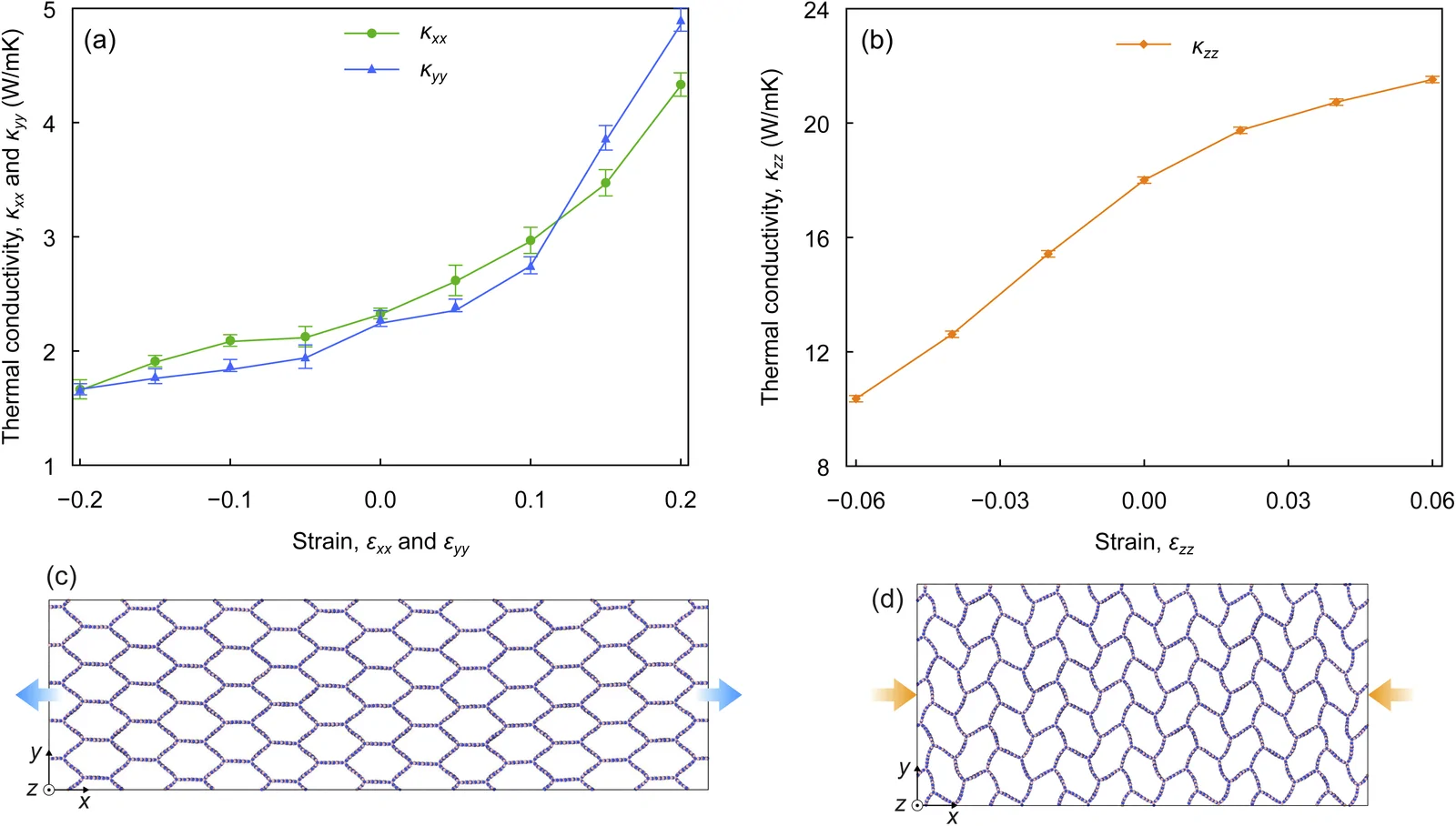
Influence of uniaxial mechanical strain on the thermal transport performance of 3D-BNHC architectures. (a) In-plane thermal conductivity (*κ*_*xx*_ and *κ*_*yy*_) as a function of tensile and compressive strains. (b) Out-of-plane thermal conductivity (*κ*_*zz*_) as a function of tensile and compressive strains. (c) Structural configuration under tensile strain, demonstrating transport-axis alignment of the honeycomb walls that minimizes junction scattering. (d) Structural configuration under compressive strain, depicting lattice buckling and structural distortion that amplifies phonon scattering and suppresses heat propagation.

Mechanical deformation offers a robust mechanism for modulating the thermal transport properties of BNHCs. Uniaxial in-plane strains (*ε*_*xx*_ and *ε*_*yy*_) ranging from −0.20 (compression) to 0.20 (tension) were applied to BNHC with *n* = 4. As illustrated in Fig. 9(a), both the in-plane thermal conductivities (*κ*_*xx*_ and *κ*_*yy*_) exhibit a strong, monotonic dependence on the applied strain, rising from approximately 1.65 W mK^−1^ at −20% compression to around 4.3 W mK^−1^ (*κ*_*xx*_) and 4.9 W mK^−1^ (*κ*_*yy*_) under 20% tension. Similarly, Fig. 9(b) shows that the out-of-plane thermal conductivity (*κ*_*zz*_) is highly sensitive to smaller strains (*ε*_*zz*_ from −0.06 to 0.06), increasing monotonically across the range. This strain-dependent behavior is primarily driven by the structural evolution of the BNHC lattice. As shown in the atomic schematic in Fig. 9(c), tensile strain promotes the straightening and alignment of the nanoribbon walls along the transport direction, minimizing geometric scattering at the structural junctions and facilitating heat flow. Conversely, as shown in Fig. 9(d), compressive strain causes the cell edges to distort and buckle, creating new phonon scattering centers that suppress thermal transport. This strain-induced modulation of phonon scattering pathways aligns with recent reports on the sensitivity of phonon transport to directional mechanical deformation.^38^ The high degree of strain sensitivity observed here highlights the potential of BNHCs for applications in advanced, strain-tunable thermal management systems.

**Fig. 10:**
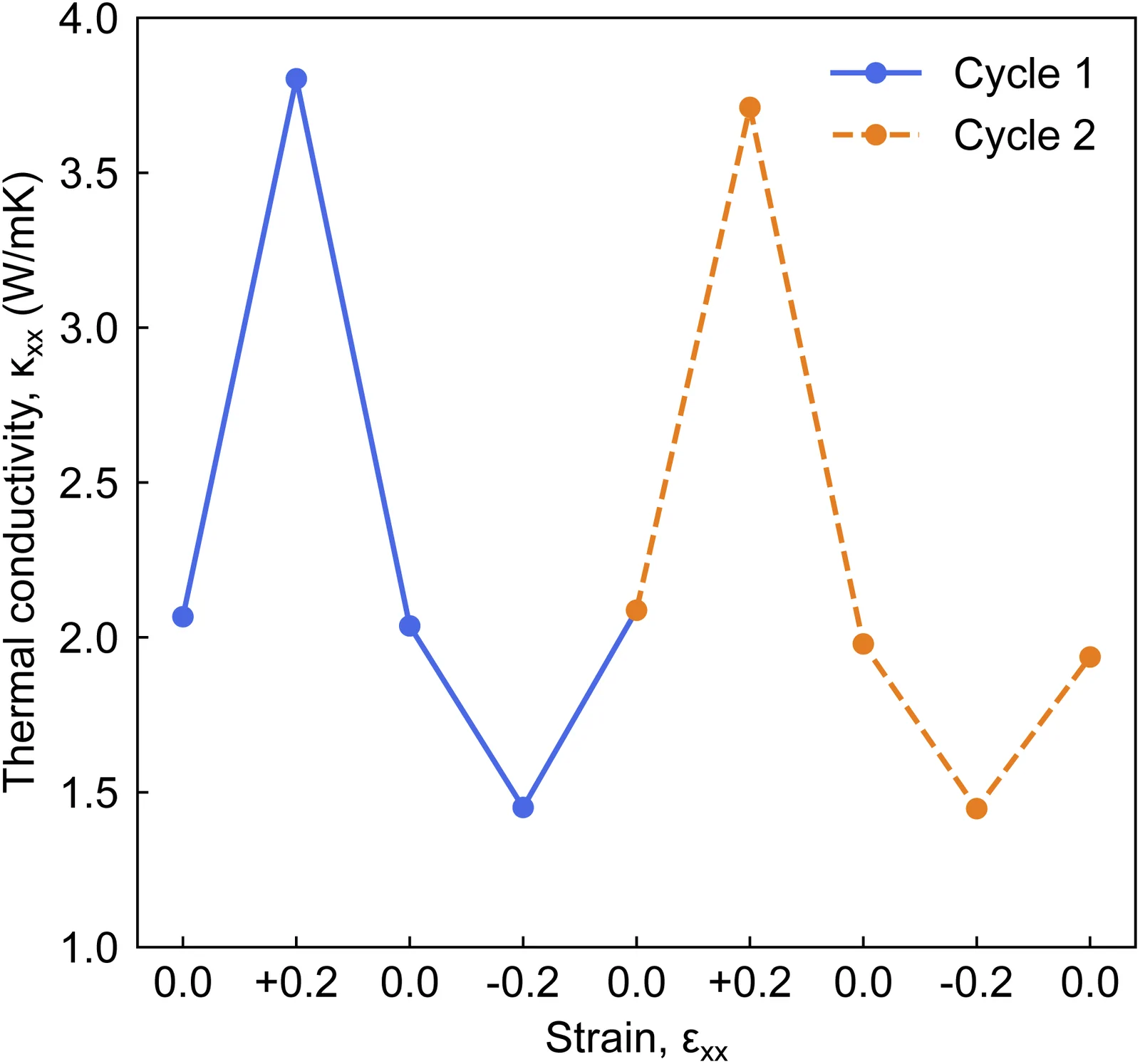
Reversible thermal conductivity (*κ*_*xx*_) of the BNHC sample under cyclic uniaxial loading–unloading along the *x*-axis (*ε*_*xx*_ ∈ [−0.20, + 0.20]) over two consecutive cycles.

To confirm the structural stability and reversibility of strain-modulated thermal transport, cyclic uniaxial loading–unloading MD simulations were conducted along the *x*-axis within a strain amplitude of *ε*_*xx*_ = ±0.20 for two complete cycles (Fig. 10). The thermal conductivity *κ*_*xx*_ exhibits fully reversible, reproducible switching behavior. Under +20% tensile strain, *κ*_*xx*_ reversibly increases from 2.08 W mK^−1^ (at zero strain) to 3.81 W mK^−1^, whereas under −20% compressive strain, *κ*_*xx*_ drops to 1.45 W mK^−1^. Upon returning to *ε*_*xx*_ = 0.0 after each state, *κ*_*xx*_ fully recovers its initial un-strained value (∼2.08 W mK^−1^ and 2.09 W mK^−1^ in cycles 1 and 2, respectively). These findings unequivocally confirm that BNHCs possess robust, highly reversible strain-tunable thermophysical properties ideal for active thermal switching applications.

### 3.4 Discussion

The ultralow thermal conductivity of BNHCs is consistent with values reported for BN aerogels.^14^ Furthermore, these architectures possess highly desirable multifunctional attributes, including low density, exceptional thermal stability, and robust mechanical strength. Unlike carbon-based architectures such as graphene aerogels, which oxidize at elevated temperatures, BNHCs are chemically inert and stable in air up to nearly 1000 °C.^39^ This exceptional thermal stability, combined with a lightweight and porous structure, makes them ideal for aerospace insulation, specifically as heat shields for re-entry vehicles, and as fire-resistant coatings that prevent heat penetration while maintaining structural integrity under extreme thermal stress. Furthermore, although hBN is intrinsically an electrical insulator, achieving ultralow thermal conductivity is a primary objective in thermoelectric research. Hybridizing BNHCs with conductive elements, such as carbon to form BNC materials, could leverage this drastic suppression of lattice thermal conductivity to significantly enhance the thermoelectric figure of merit.^40^

**Fig. 11:**
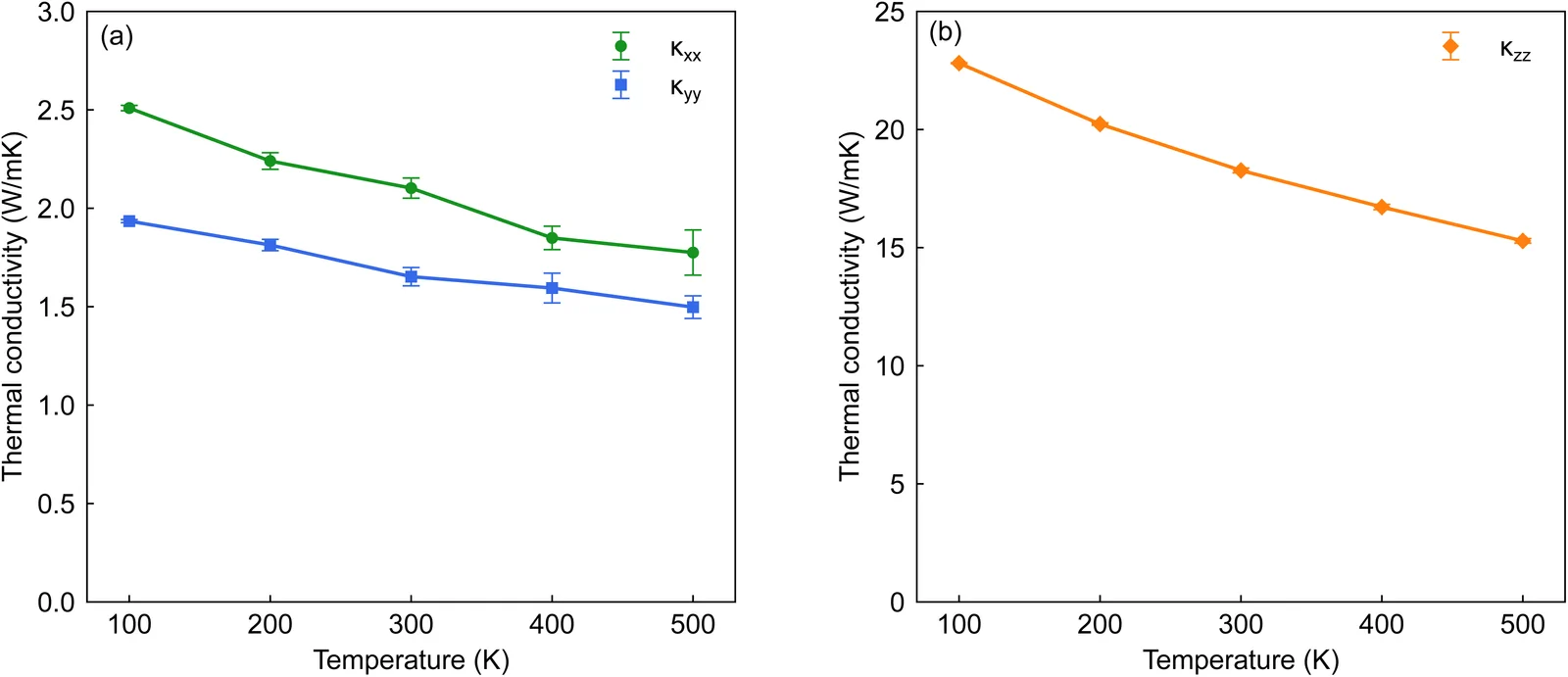
Temperature dependence of thermal conductivity components for the SG BNHC sample across the range of 100 K ≤ *T* ≤ 500 K. (a) In-plane thermal conductivity along the *x*-axis (*κ*_*xx*_) and *y*-axis (*κ*_*yy*_). (b) Out-of-plane thermal conductivity along the *z*-axis (*κ*_*zz*_, orange line). Error bars denote standard deviations obtained from molecular dynamics simulations.

To further clarify the thermophysical properties of BNHCs, we investigated the temperature-dependent thermal conductivity of the SG sample in the range of 100 K ≤ *T* ≤ 500 K, as depicted in Fig. 11. Across all directions (*κ*_*xx*_, *κ*_*yy*_, and *κ*_*zz*_), the thermal conductivity monotonically decreases with increasing temperature. Specifically, as temperature rises from 100 K to 500 K, *κ*_*xx*_ drops from 2.51 to 1.78 W mK^−1^, *κ*_*yy*_ decreases from 1.94 to 1.50 W mK^−1^ (Fig. 11(a)), while *κ*_*zz*_ declines from 22.8 to 15.3 W mK^−1^ (Fig. 11(b)). This inverse temperature dependence (*κ* ∝ 1/*T*) indicates that intrinsic phonon–phonon scattering is the predominant mechanism governing thermal transport in this temperature range.

To contextualize the potential of 3D-BNHCs for high-temperature thermal insulation and mechanical protection, we compare their transport and mechanical metrics against state-of-the-art cellular and porous materials. The in-plane thermal conductivity achieved here (<5 W mK^−1^ for *n* ≥ 3) is comparable to ultralight BN aerogels (∼0.02–2.0 W mK^−1^)^14^ and vastly lower than bulk hBN (∼300–750 W mK^−1^). Crucially, while carbon aerogels exhibit oxidative degradation at temperatures above 500 °C (773 K),^13^ sp^2^-bonded hBN frameworks maintain structural and oxidation resistance in air up to nearly 1000 °C,^39^ confirming their thermal stability in extreme environments. Furthermore, from an energy dissipation standpoint, the compressive stress plateaus yield high specific energy absorption values, outperforming conventional macroscopic foams of equivalent mass density. This combination of ultralow thermal conductivity, high-temperature oxidation resistance, and buckling-driven energy absorption provides direct quantitative support for deploying 3D-BNHCs in extreme thermal shielding and mechanical damping applications.

Beyond serving as a specific case study for boron nitride, the fundamental transport mechanics revealed here demonstrate broader chemical relevance across sp^2^-hybridized covalent networks and 3D porous architectures. From a structural chemistry perspective, seamlessly interconnecting 2D nanoribbons *via* monatomic triple-line junctions provides a general topological design rule applicable to carbon networks, BNC heterostructures, and group-IV monochalcogenides. Crucially, this seamless covalent assembly preserves the local sp^2^ bonding environment and coordination sphere, leaving the network free of dangling bonds or under-coordinated defect sites. This pristine defect chemistry accounts for the exceptional environmental and oxidative stability of 3D-BNHCs, overcoming the severe high-temperature degradation typical of carbon-based analogues. Furthermore, the extreme suppression of lattice thermal conductivity (*κ* < 5 W mK^−1^) without introducing chemical point defects offers a strategic framework for energy materials design. For instance, targeted chemical synthesis can leverage this topology in mixed-element systems, such as ternary BNC networks, to decouple electronic and thermal transport, effectively suppressing phonon propagation while preserving electronic pathways for advanced thermoelectric applications.

## 4 Conclusion

In summary, we have systematically investigated the thermal transport, mechanical performance, and strain-tunable characteristics of 3D boron nitride honeycomb (3D-BNHC) architectures using non-equilibrium molecular dynamics simulations. Our findings demonstrate that these topologies exhibit an ultralow in-plane thermal conductivity (<5 W mK^−1^), representing a two-order-of-magnitude reduction compared to pristine 2D hBN, which spectral conductance analysis and spatial heat flux vector mapping reveal is driven by intense phase-boundary scattering at the junction lines alongside localized, closed heat flux patterns that trap heat carriers within the expanded cell cores. Furthermore, the network exhibits a highly pronounced spatial thermal anisotropy; out-of-plane transport (*K*_*zz*_) scales nearly linearly with relative mass density (*α* ≈ 0.90) due to the continuous, unbroken zigzag nanoribbons, whereas in-plane transport follows a macroscopic power-law scaling (*α* ≈ 1.5) characteristic of complex porous networks. Mechanically, the 3D-BNHC structures preserve excellent structural integrity while introducing ultralight weight properties, displaying a stretch-dominated, brittle catastrophic failure under tension with near-unity scaling exponents for both Young's modulus and ultimate tensile strength, while transitioning to a classic multi-stage cellular deformation under compression where lower-density configurations yield *via* cooperative, continuous cell-wall buckling to generate long, flat stress plateaus ideal for progressive, rupture-free energy absorption. Finally, we demonstrate that mechanical deformation offers a highly efficient route for the dynamic tuning of heat transport, enabling a reversible modulation of in-plane thermal conductivity that is mechanistically governed by a competition between transport-axis alignment under tensile extension and lattice buckling under compressive load. Given their combination of ultralow thermal conductivity, strong mechanical resilience, and exceptional chemical inertness at elevated temperatures, these 3D-BNHC architectures hold immense promise as multi-functional material candidates for high-performance thermal insulation in extreme environments, protective mechanical dampers, and strain-controlled thermal routing systems.

## Conflicts of interest

There are no conflicts to declare.

## Supplementary Material

RA-016-D6RA04965F-s001

## Data Availability

The data supporting this article have been included as part of the supplementary information (SI). Supplementary information: includes additional figures and tables. See DOI: https://doi.org/10.1039/d6ra04965f.
