# Supplementary material for: Topological control of mechanical resilience and tunable thermal transport in 3D boron nitride honeycombs
Source: RSC Adv. 2026 Aug 11;16(40):46784–97. doi: 10.1039/d6ra04965f (PMC13459184; doi:10.1039/d6ra04965f)
Supplement: Supplementary file 1 [file RA-016-D6RA04965F-s001.pdf]

Supplementary Information (SI) for RSC Advances.
This journal is © The Royal Society of Chemistry 2026

Supplement Information
Topological Control of Mechanical Resilience and Tunable Thermal Transport in 3D Boron Nitride Honeycombs

Minh-Quan Doan[1], Tuan-Dung Nguyen[1], Tuan Nguyen Van[2], Douglas Soares Galvão[3], Manh-Huong Phan[1], and Le Van Lich*[1,4]

[1]Center for Materials Innovation and Technology, VinUniversity, Hanoi, Vietnam
[2]Department of Physics, Le Quy Don Technical University, Hanoi, Vietnam
[3]Department of Applied Physics, State University of Campinas, 13083-970, Campinas, São Paulo, Brazil
[4]College of Engineering and Computer Science,VinUniversity, Hanoi, Vietnam

*Email: lich.lv@vinuni.edu.vn

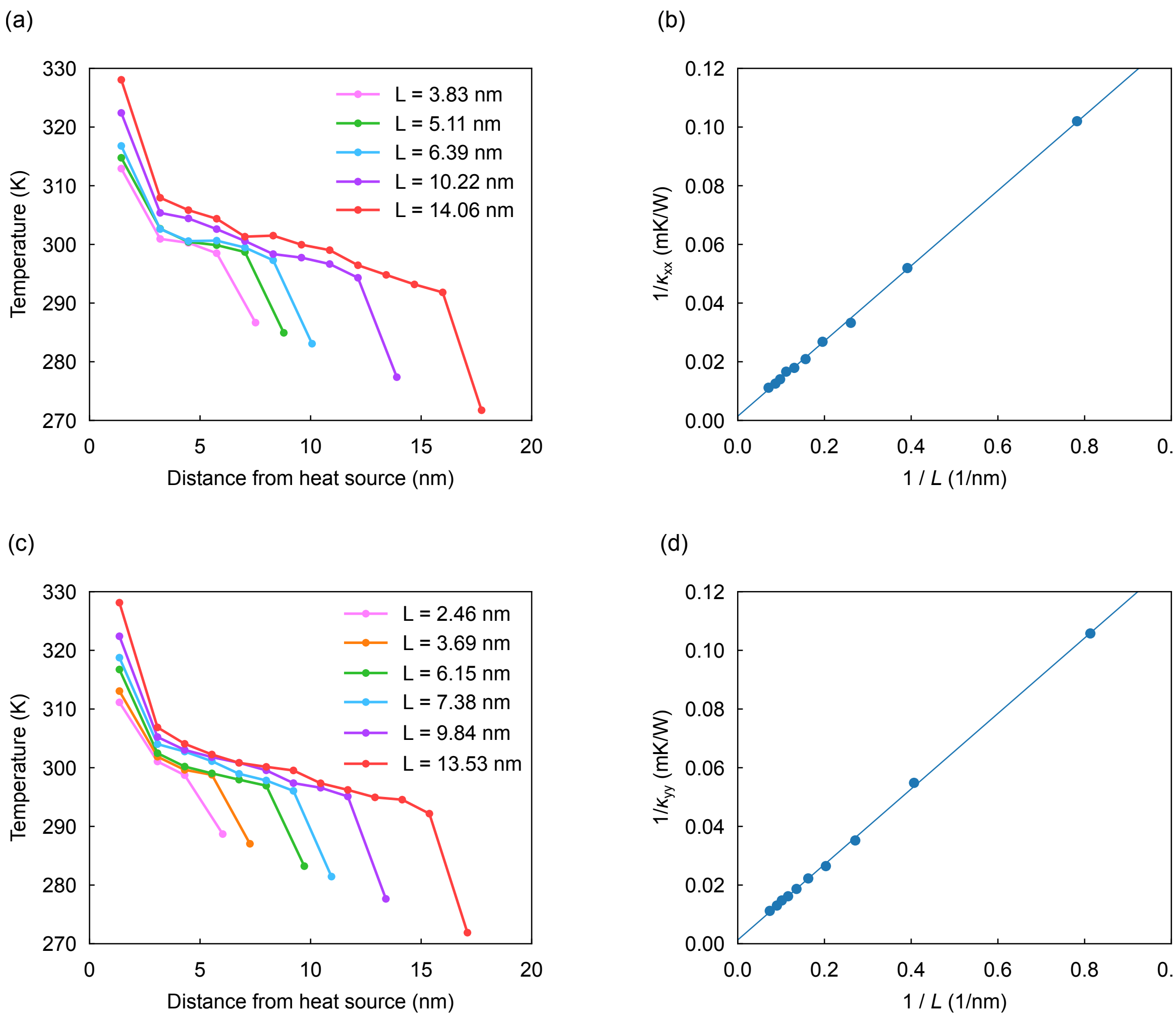

Figure S1: Thermal conductivity of 2D Boron Nitride: (a, c) Steady-state temperature distribution for with lengths (L) ranging from 2 nm to 14 nm, along x and y direction, respectively. (b, d) Extrapolation of bulk thermal conductivity using the linear relationship between inverse system length ($1/L$) and inverse thermal conductivity ($1/\kappa_{xx}$, $1/\kappa_{yy}$).

Table S1: Length, width and thickness (model size) of Boron Nitride Honeycomb model during NEMD simulation in x, y, z directions

NEMD direction	n	L (Å)	w (Å)	t (Å)
$\boldsymbol{x}$	1	70,87,104,121,155,172,206,240	98.4	86.1
	2	76,106,136,165,195,225,255,285,315,374	86.1	86.1
	3	108,151,193,236,278,321,364,406,449,534	123.0	98.4
	4	140,195,251,306,417,583	159.9	147.6
	5	172,240,308,376,513	196.8	147.6
$\boldsymbol{y}$	1	109,149,188,228,267,306,346	85.2	86.1
	2	87,122,156,191,225,259,294,328,363,432	89.5	86.1
	3	124,173,223,272,321,370,419,469,518,616	127.8	98.4
	4	161,225,289,353,481,673	166.1	147.6
	5	198,277,355,434,592,828	204.5	147.6
$\boldsymbol{z}$	1	50,70,89,108,129,168,246	68.2	68.9
	2	50,70,89,108,129,168,246	119.3	120.5
	3	50,70,89,108,129,168,246	170.4	172.2
	4	50,60,70,80,84,89,129	166.1	159.9
	5	50,60,70,80,84,89,129	204.5	196.8

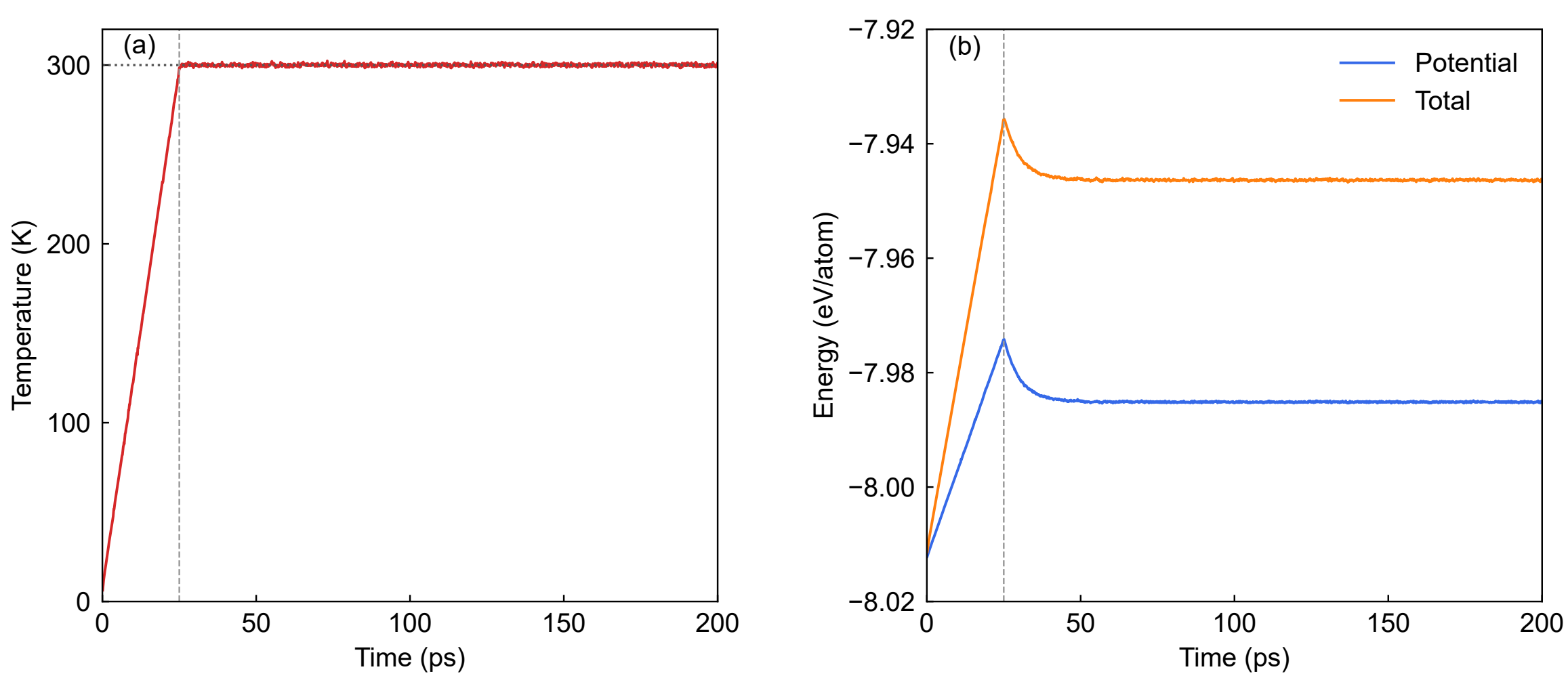

Figure S2: Thermal equilibration and energy convergence profiles from molecular dynamics simulation: (a) Temperature trajectory and (b) time evolution of potential energy and total energy in eV/atom.
